# Renal Artery Catheterization for Microcapsules’ Targeted Delivery to the Mouse Kidney

**DOI:** 10.3390/pharmaceutics14051056

**Published:** 2022-05-14

**Authors:** Olga I. Gusliakova, Ekaterina S. Prikhozhdenko, Valentina O. Plastun, Oksana A. Mayorova, Natalia A. Shushunova, Arkady S. Abdurashitov, Oleg A. Kulikov, Maxim A. Abakumov, Dmitry A. Gorin, Gleb B. Sukhorukov, Olga A. Sindeeva

**Affiliations:** 1 Science Medical Center, Saratov State University, 83 Astrakhanskaya Str., 410012 Saratov, Russia; prikhozhdenkoes@sgu.ru (E.S.P.); voplastun@gmail.com (V.O.P.); oksanaamayorova@gmail.com (O.A.M.); shushunovan.a@gmail.com (N.A.S.); 2 Center for Neurobiology and Brain Restoration, Skolkovo Institute of Science and Technology, 3 Nobel Str., 143005 Moscow, Russia; a.abdurashitov@skoltech.ru (A.S.A.); g.sukhorukov@skoltech.ru (G.B.S.); 3 Institute of Medicine, National Research Ogarev Mordovia State University, 68 Bolshevistskaya Str., 430005 Saransk, Russia; oleg-kulikov-84@mail.ru; 4 Department of Medical Nanobiotecnology, Pirogov Russian National Research Medical University, 1 Ostrovityanova Str., 117997 Moscow, Russia; abakumov_ma@rsmu.ru; 5 Center for Photonics and Quantum Materials, Skolkovo Institute of Science and Technology, 3 Nobel Str., 143005 Moscow, Russia; d.gorin@skoltech.ru; 6 School of Engineering and Materials Science, Queen Mary University of London, Mile End Road, London E1 4NS, UK

**Keywords:** renal artery catheterization, polyelectrolyte microcapsules, targeted delivery, mouse kidney, biodistribution, migration, blood flow, optoacoustic mesoscopy, laser speckle contrast imaging

## Abstract

The problem of reducing the side effects associated with drug distribution throughout the body in the treatment of various kidney diseases can be solved by effective targeted drug delivery. The method described herein involves injection of a drug encapsulated in polyelectrolyte capsules to achieve prolonged local release and long-term capillary retention of several hours while these capsules are administered via the renal artery. The proposed method does not imply disruption (puncture) of the renal artery or aorta and is suitable for long-term chronic experiments on mice. In this study, we compared how capsule size and dosage affect the target kidney blood flow. It has been established that an increase in the diameter of microcapsules by 29% (from 3.1 to 4.0 μm) requires a decrease in their concentration by at least 50% with the same suspension volume. The photoacoustic method, along with laser speckle contrast imaging, was shown to be useful for monitoring blood flow and selecting a safe dose. Capsules contribute to a longer retention of a macromolecular substance in the target kidney compared to its free form due to mechanical retention in capillaries and slow impregnation into surrounding tissues during the first 1–3 h, which was shown by fluorescence tomography and microscopy. At the same time, the ability of capillaries to perform almost complete “self-cleaning” from capsular shells during the first 12 h leads to the preservation of organ tissues in a normal state. The proposed strategy, which combines endovascular surgery and the injection of polymer microcapsules containing the active substance, can be successfully used to treat a wide range of nephropathies.

## 1. Introduction

Mechanized, thin, and complex catheters and endovascular devices have become a fundamentally new approach in targeted delivery in nephrology [1,2,3,4]. Carrying out this procedure under local anesthesia made this manipulation widely available for all categories of patients [5,6,7].

Targeting drugs [7,8], contrast agents [9,10], biomaterials [11,12,13], stem cells [14], and drug delivery systems [15,16] by injection into the renal artery has confirmed the effectiveness of local targeting to the kidney in many studies. However, mainly two variants of using this technique have reached real clinical practice: the introduction of contrast agents (for diagnostics, namely angiography [4,17]) and irreversible embolization (preliminary procedures before total nephrectomy [18,19]) or chemoembolization [20].

The limitations of using biomaterials and drug delivery systems in the clinic are associated with the risk of embolization, not only of the targeted kidney, but also of other vital organs [21]. Unfortunately, most research papers are limited to describing the effectiveness of intra-arterial targeting, and only a few are devoted to the need to select safe strategies for this procedure [14,22].

Choosing the right strategy requires sufficiently detailed and extensive studies in animal models. Note that the rodent model is the most accessible to a wide range of researchers, but the size of the vessels is a significant limitation. Incorrectly performed renal artery injection techniques can not only disrupt the blood supply to the organ itself, but also lead to less pronounced damage [15,23,24], which will further distort the result of the therapy effectiveness study. A correctly performed procedure is the key to studying the mechanisms of drug and drug carrier interaction with the renal vascular system, assessing their migration and the rate of drug diffusion into tissues. All this is necessary for the correct choice of the drug container type and composition for each specific disease and physiological condition.

Polyelectrolyte microcapsules are one of the promising systems for local prolonged release of drugs. There are a number of unique properties that make them very suitable candidates for arterial targeting of the kidneys. Firstly, they have a short circulation time; most of the dose accumulates in the vessels during the first minute at a capsule size of 3–5 μm [25,26]. They are less effective when administered intravenously due to the first passage through the vessels of the lungs and mechanical accumulation in them [25,27]. However, this is a significant advantage for arterial targeting, as most of the dose “entangles” in the branched vessels of the target organ, through which it first passes [22,27]. Secondly, microcapsules can be mechanically deformed due to the absence of a rigid core, which allows maintaining blood flow in the vessels in which the capsules have accumulated [28,29]. Thirdly, capsules based on polyarginine and dextran sulfate are biocompatible and non-toxic [30,31], highly stable [27], and completely biodegradable within a few weeks [32]. Fourthly, if necessary, they can be further modified with fluorescent [33] and photodynamic [34] dyes or gold [35] or magnetite [26,36,37,38,39,40] nanoparticles. Polyelectrolyte microcapsules are suitable for the encapsulation of biologically active substances, such as NGF [41], sodium-channel blocker QX-314 [42], mRNA, and siRNA [43].

The method of implanting a catheter into the mouse renal artery by a small puncture in the femoral artery and the criteria for evaluating the correctness of each manipulation step are described here in detail. This method is similar to the clinical approach [44] and does not involve long-term blockade of the blood flow of the target organ [15] or disruption of the integrity of the abdominal aorta or renal artery [14,45], unlike other methods. In addition, the features of the use of polyelectrolyte microcapsules as a promising system for the delivery of macromolecular cargo to the target kidney through the renal artery is shown here. The impact of microcapsule size and concentration on the blood flow of the target kidney is investigated with laser speckle imaging of blood flow and photoacoustic tomography. Comparison of the biodistribution of free and encapsulated macromolecular cargo over time is demonstrated. The final part of the article is devoted to the trend of cargo accumulation and capsular shells’ migration in the first hours after injection through the renal artery.

## 2. Materials and Methods

### 2.1. Materials

Calcium chloride (dihydrate), sodium carbonate (anhydrous), ethylenediaminetetraacetic acid disodium salt (EDTA, dihydrate), dextran sulfate sodium salt (DsS, MW > 70,000), poly-L-arginine hydrochloride (pArg, MW > 70,000), Rhodamine B isothiocyanate (RITC), phosphate-buffered saline (PBS, 0.01 M), bovine serum albumin (BSA, lyophilized powder), trypsin from porcine pancreas (∼1500 U/mg), and tris(hydroxymethyl)aminomethane (≥9.8%) were purchased from Sigma-Aldrich (Taufkirchen, Germany). Hydrochloric acid was obtained from Reakhim (Moscow, Russia); dimethyl sulfoxide (DMSO) was purchased from Merck (Darmstadt, Germany); cyanine 7 NHS ester (Cy7) was obtained from Lumiprobe (Moscow, Russia). All chemicals were used without further purification. Deionized water produced with a water treatment system Milli-Q (Merck Millipore, Darmstadt, Germany) was used in all experimental stages.

### 2.2. Microcapsules Labeled by Cyanine 7 and RITC: Preparation and Characterization

#### 2.2.1. Preparation of Cy7-Conjugated BSA

First, 60 mg of BSA was dissolved in 13.5 mL of 0.1 M PBS buffer (pH 8.3). Cy7 (5 mg) was dissolved in anhydrous DMSO (1.5 mL). After that, the Cy7 solution was added to a BSA solution (15 mL, 4 mg/mL, PBS buffer, pH 8.3). The mixture was stirred overnight at 4 °C temperature. Cy7 was conjugated with BSA solution washed from excess reagents by extensive dialysis in water. The amount of the encapsulated Cy7 dye into microcapsules was calculated from spectroscopy measurements by the analysis of dye remaining in the supernatants after each core washing step during the encapsulation process.

#### 2.2.2. Preparation of RITC-Conjugated BSA

BSA-RITC was prepared according to the procedure described by Denis V. Voronin et al. [28]. Briefly, a BSA solution (2 mg/mL) in PBS buffer (pH 8) was incubated with an RITC solution in ethanol (5 mg/mL) at 4 °C in the dark within 12 h. Finally, freshly prepared RITC-conjugated BSA was dialyzed against deionized water at 4 °C in the dark for 3 days.

#### 2.2.3. Microcapsules’ Preparation

The vaterite microspheres (CaCO_3_) were used as templates to form microcapsules using a layer-by-layer technique, as described in [46], with some adjustments. The vaterite templates were prepared by mixing equimolar solutions of two salts. Briefly, equal amounts (0.615 mL of 1 M aqueous solutions) of CaCl_2_ and Na_2_CO_3_ salts were added to 2.0 mL BSA-Cy7 solution (2 mg/mL) and 0.5 mL water. The crystallized microparticles with incorporated fluorescent conjugate BSA-Cy7 were sedimented by centrifugation at 1000 rcf and washed with deionized water three times. The polyelectrolyte layers’ formation was carried out by cascade adsorption of pArg and DsS with triple washing of each layer with deionized water. The second layer of DsS was replaced with BSA-RITC. Layering continued until 3 bilayers were reached. The complete shell composition was pArg/DsS/pArg/BSA-RITC/pArg/DsS. Then, the vaterite cores were dissolved in 0.2 M EDTA (pH 7.3) to form hollow microcapsules. EDTA can gently dissolve calcium carbonate microparticulate cores and leave the polymer shell intact. Microcapsules with a size of 3.1 μm and 4.0 μm were obtained to determine the effect of size on the restoration of blood flow after their injection. The size of microcapsules differs little from the size of calcium carbonate microparticles, on the basis of which the capsules were synthesized, since the thickness of the polymer shell usually does not exceed ∼50 nm [22].

#### 2.2.4. Confocal Laser Scanning Microscopy

Fluorescent properties of as-prepared microcapsules were studied with a Leica TCS SP8 X inverted confocal microscope (Leica Microsystems, Wetzlar, Germany) equipped with a white light pulsed laser source (1.5 mW laser power) focused through a 20 × /0.70 N.A. objective. The following settings were used: 554 nm excitation with 570–650 nm emission detection (RITC), 670 nm excitation with 700–795 nm emission detection (Cy7). The size distribution of microcapsules was evaluated using CLSM images of 200 microcapsules.

### 2.3. Animal Studies

The laboratory animals were treated according to the rules of Saratov State Medical University (Ethics Committee Protocol No. 7, dated 2 February 2021) and the Geneva Convention of 1985 (International Guiding Principles for Biomedical Research Involving Animals). All experimental procedures were performed on white Balb/c male mice 6–8 weeks old (20–25 g weight) using general anesthesia (Zoletil mixture (40 mg per kg, 50 μL, Virbac SA, Carros, France) and 2% Rometar (10 μL and 10 mg per kg, Spofa, Czech Republic)) via intraperitoneal injection. At the end of the experiment, the animals were euthanized by an overdose of anesthesia.

#### 2.3.1. Blood Flow Response to Capsules’ Administration

The evaluation of changes in the left kidney blood flow after the injection of microcapsules was carried out in vivo in real-time on a self-made laser speckle contrast imaging (LSCI) system. To do this, after a small incision was made on the back of the mouse parallel to the spine in the region of the left kidney, the surrounding tissues were pushed apart and fixed. Measurements were performed under general anesthesia and directly from the surface of the left kidney before and 15 min and 24 h after microcapsules’ injection into the renal artery. The collection of blood flow data began after the hemodynamic parameters were balanced. Blood flow alteration was evaluated in 6 groups of mice: animals without injection (control group), after administration of saline solution (sham-operated group), 3.1 μm capsule suspension in two dosages: 20 × 10${}^{6}$ and 30 × 10${}^{6}$ capsules (2 experimental groups), and 4.0 μm capsule suspension in two dosages: 10 × 10${}^{6}$ and 20 × 10${}^{6}$ capsules (2 experimental groups). Blood flow values measured before injection were considered as baseline values and calculated for each individual mouse. All blood flow values were normalized by individual baselines, and alterations were calculated as percentages.

#### 2.3.2. Laser Speckle Contrast Imaging System

Laser speckle contrast imaging (LSCI) is a powerful non-contact and non-destructive optical modality that allows for qualitative measurement of blood flow in superficial vessels [22,47,48].

Irradiation from a Thorlabs CPS635S laser diode with the help of a ground glass diffuser (Thorlabs DG10-220-MD) and a converging lens (Thorlabs LA1131-ML) uniformly illuminated the surface of the targeted kidney. To capture the subjective speckle pattern, the digital camera Basler a2A2600-64ucBAS and the Navitar MVL25M1 photo-lens were utilized. Analyzing the spatial spectrum of the speckle pattern, the lens F-stop was tuned to satisfy the Nyquist criterion. Linear polarizer was placed in front of the photo-lens and adjusted to be in line with the polarization of the laser source. The exposure time and frame rate of the digital camera were set to 10 ms and 30 frames per seconds, respectively. Custom-made Python-based software was used to control the LSCI system and process the captured speckle images. Spatial speckle contrast was calculated as the ratio between the standard deviation of the pixel’s intensity and their mean value in a 5 × 5 sliding window [49]. To increase the signal-to-noise ratio of the computed speckle contrast images, 25 consecutive speckle contrast frames were averaged. Each blood flow map was smoothed by a Gaussian filter with sigma equal to 7 in the spatial dimension to eliminate high-frequency oscillations and leave only coarse blood flow changes. For each dataset, an ROI of 200 × 200 pixels was selected; the mean and standard deviation were calculated in the ROI. Data processing was carried out using the NumPy v.1.16.5. and SciPy v.1.3.1. packages of Python Base Version 3.6.

#### 2.3.3. Photoacoustic Visualization of Kidney’s Blood Flow

The study of the impact of microcapsules’ administration on the blood flow in the kidney’s vessels was performed using a mesoscope (RSOM Explorer P50, iThera Medical, München, Germany) in epi-illumination mode. The RSOM is equipped with a nanosecond pulsed laser (pulse duration 2.5 ns, repetition rate 1 kHz, pulse energy 1 mJ at a wavelength 532 nm). The ultrasonic signal excited in the biological tissue was captured using a LiNbO_3_-based spherically focused transducer (center frequency 50 MHz, bandwidth 11–99 MHz).

Visualization of the kidney’s vasculature was made before and 5, 15 min, 1 h, 24 h, and 5 days after the capsules’ administration. Two doses of 10 × 10${}^{6}$ (safe) and 20 × 10${}^{6}$ (unsafe) were selected to demonstrate the effect of reducing blood flow with a harmful dosage of 4.0 μm capsules. Directly prior to measurement, each animal was anesthetized, then transferred to a heated stage (37 °C) of the mesoscope. The left kidney was cleared from the surrounding tissues to allow direct imaging of this organ. The source of laser radiation and the ultrasonic transducer were located in the center of the left kidney slightly above its surface. The gap between the surface of the left kidney and the transducer was filled with ultrasound gel (see Supplementary Materials, Figure S1). The size of the scanning area was chosen as 4 × 4 mm.

Image post-processing was carried out using rLabs_v1.19.04 software. The signal accumulated by the transducer was divided into two ranges: low-frequency (11–33 MHz) and high-frequency (33–99 MHz). This approach helped to separate objects by size. Thus, a low-frequency signal represents larger objects (red color in the images), and a high-frequency signal represents smaller objects (green color). Using rLabs_v1.19.04, quantitative data were obtained on the intensity of the detected signal in two ranges in the manually selected ROI. The alteration of the acoustic signal for every time point and frequency band was calculated as a percentage of the intensity of the detected acoustic signal in a certain frequency range at a certain time point time to the intensity of the acoustic signal in the same frequency range before the of capsules’ injection:

$$A=\frac{I_{t}^{f}}{I_{0}^{f}}\cdot 100\%,$$

where *A* is the alteration of the acoustic signal; $I_{0}$ and $I_{t}$ are the intensities of the acoustic signal before injection and at a specified time point, respectively; *f* is the frequency band.

Furthermore, photoacoustic images of the vessels of the left kidney at different depths were obtained by splitting the 3D reconstruction into separate layers using the free software ImageJ [50].

#### 2.3.4. Microcapsules’ Biodistribution

The IVIS SpectrumCT In Vivo Imaging System (PerkinElmer, Waltham, MA, USA) was used to determine the fluorescent marker Cy7 distribution in the mouse body after renal artery injection. Excitation and emission were set at 745 and 800 nm, respectively. Post-processing was performed via Living Image software v.4.7.3.

The kinetics of the fluorescence redistribution in mouse organs was observed for 24 h. For this purpose, 10 × 10${}^{6}$ capsules with a 4.0 ± 0.6 μm size or corresponding amount of free BSA-Cy7 conjugate were injected into the left renal artery. After 1, 15 min, 1, 3, 6, and 24 h, animals with both types of administration were sacrificed, and their organs (kidneys, lungs, heart, liver, spleen, stomach, intestines, appendix), as well as the organs of mice without any injection (control group) were imaged ex vivo.

Total Radiant Efficiency (TRE) for each organ was measured within their boundaries. TRE expresses total photon amount per second from the selected area with normalizing for illumination intensity. The percentage of injected dose (%ID) of each organ was calculated as follows:

$$\%ID_{organ}=\frac{TRE_{organ}}{\sum_{all\;organs}TRE}\cdot 100\%$$

$$TRE_{organ}=TRE_{experimental}-\frac{TRE_{control}}{Area_{control}}\cdot Area_{experimental}$$

where $TRE_{organ}$ is total radiant efficiency for a certain organ, $TRE_{experimental}$ is the measured TRE value of a certain organ from the group with any injection, $TRE_{control}$ is the measured TRE value of a certain organ from the group without any injection, $Area_{experimental}$ is the area of a certain organ from the group with any injection, $Area_{control}$ is the area of a certain organ from the group without any injection, and $\sum_{all\;organs}TRE$ is the sum of all $TRE_{organ}$ values for one mouse. The second term on the right of $TRE_{organ}$ reflects the autofluorescence of a certain organ. All $TRE_{organ}$ values showed below are expressed as the mean ± standard deviation (n = 3).

#### 2.3.5. Microcapsules’ Localization in the Kidney Tissue

To investigate the microcapsules’ localization in kidney tissues, a cryosection study was applied. Two microcapsule dosages (10 × 10${}^{6}$ and 20 × 10${}^{6}$, 4.0 μm) were injected via the left renal artery. The left kidneys were placed in a 10% formalin solution for at least 24 h. Then, the organs were submerged in different solutions prior to cryosectioning: PBS buffer (for 7 days); 10, 20, 30 *w/v*% sucrose solutions in PBS buffer (for 2 h each). The cryosectioning procedure was performed with a Leica cm1950 cryostat (Wetzlar, Germany).

Prepared cryosection samples (30 μm thickness) were investigated using a Leica TCS SP8 X confocal microscope. A 405 nm diode source was used to excite the renal tissue’s autofluorescence (415–500 nm detection), and a 554 nm laser line of white light laser was used to excite RITC fluorescence in injected microcapsules (570–650 nm detection).

#### 2.3.6. Histological Studies

Kidneys were subjected to histological examination 120 h (5 days) after left renal artery injection of microcapsules in doses of 10 × 10${}^{6}$ and 20 × 10${}^{6}$ microcapsules with a size of 4.0 ± 0.6 μm in 10 μL saline solution. Tissue samples were fixed in neutral formalin. Then, the samples were desiccated by dehydration in isopropyl alcohol and embedded in paraffin. The 5 μm-thick slides were stained with hematoxylin and eosin (H & E staining). Morphologic analysis of the histological samples was performed using an Olympus digital image analysis system.

#### 2.3.7. Quantification of Fluorescent Dye Content in Kidneys

To estimate capsules or the BSA/Cy7 amount delivered to the targeted organ, the enzymatic degradation of both kidneys was carried out in accordance with our previous study [22]. Animals were euthanized by an overdose of anesthesia with both kidneys sampled 1, 15 min, 1, 3, 6, and 24 h after dye or microcapsules’ injection. Then, the organs were homogenized, placed into separate tubes, and heated in a thermoshaker at 37 °C, and trypsin solution (800 μL, 10 mg/mL in 50 mM Tris-HCl buffer, pH 8.0) was added to perform enzymatic degradation of the kidneys. The obtained mixture was left for 2 h under the following conditions: 1000 rpm, 37 °C. To prepare the calibration solutions, intact kidneys and known dye/microcapsules concentrations were used in the same approach. After 2 h of continuous shaking, mixtures were centrifuged (2500 rpm, 1 min), diluted with water in a 1:1 ratio, and placed into 5 wells of a 96-well plate.

Emission spectra (745 nm excitation, 770–840 nm emission range with 1 nm step) were collected with a CLARIOstar Plus microplate reader (BMG Labtech, Ortenberg, Germany). The emission intensity at 785 nm was used in the calibration and calculation of the injected dose percentage in both kidneys. The percentage of injected dose was calculated as follows:

$$\%ID=\frac{Mass_{in\;sample}^{BSA-Cy7}}{Mass_{injected}^{BSA-Cy7}}\cdot 100\%$$

where $Mass_{in\;sample}^{BSA-Cy7}$ is the amount of fluorescence conjugate in the kidney estimated according to the calibration line and $Mass_{injected}^{BSA-Cy7}$ is the total amount of fluorescence conjugate injected to the left kidney in encapsulated or free form.

## 3. Results

### 3.1. Minimally Invasive Mouse Renal Artery Catheterization Technique

**Figure 1:**
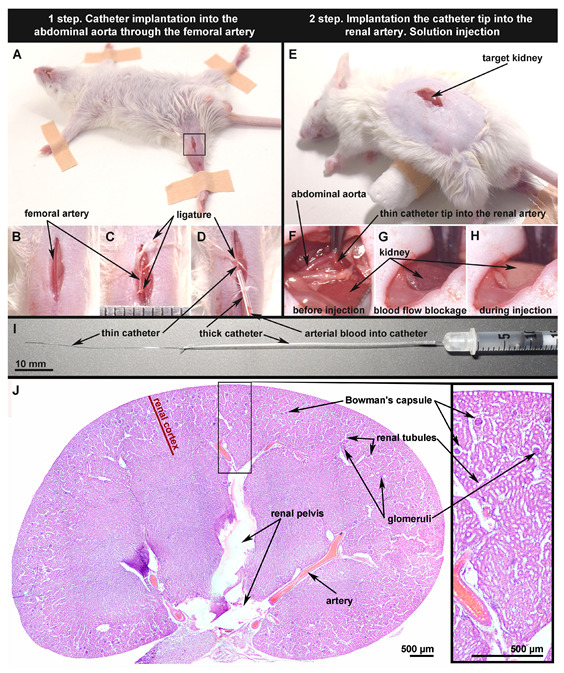
Main stages of the minimally invasive mouse renal artery catheterization technique (**A**–**H**) using polyurethane tubing (**I**); skin incision on the right thigh for access to the femoral artery marked with a black square (**A**). Renal artery before (**B**) and after ligation (**C**). The thin part of the catheter (4.5 cm, inner diameter 0.25 mm) is completely implanted in the renal artery. The arterial blood in the catheter indicates that implantation was performed correctly (**D**). Skin incision in parallel with the spine above the kidney for access to the abdominal aorta and renal artery (**E**). Catheter tip pushed into the renal artery through the abdominal aorta; the artery’s blood flow is preserved, and the targeted kidney has a normal color (**F**). Renal artery walls are tightly pressed to the catheter walls using tweezers to prevent the entering of injection solution into the aorta; the kidney has a dark color due to the blood flow blockage (**G**). The kidney color change from dark to light during injection indicates that the manipulation was performed correctly (**H**). The subgross histological image of the target kidney 5 days after the saline injection (sham operation) shows that the kidney tissue has a normal structure. The thickness of the histological samples is 5 μm; dyes: hematoxylin and eosin (H & E) (**J**).

The efficiency and safety of targeting the drugs in free or encapsulated form to the kidney via the renal artery largely depends on the correctness of the procedure for their injection. This is especially important for experimental work with small rodents due to the limited or impossibility of using non-invasive methods for detecting the position of catheters and delivery devices while they move through the vessels. In this regard, at the first stage of the study, we worked out a method for delivering a catheter to the mouse renal artery (Figure 1A–H), similar to that used in real clinical practice.

The first stage of the operation begins with the catheter implantation into the mouse femoral artery. For this, the animal must lie on its back (Figure 1A). Hair on the mouse thigh should be removed. This will help clearly identify the femoral vein and artery and make a small skin incision (5–6 mm) above them (Figure 1A,B). Note that the femoral vein is always located closer to the tail and the femoral artery further. In addition, the artery usually has a lighter color. After artery separation from the surrounding tissues, two ligatures were applied to it: upper and lower (Figure 1C). The lower ligature blocks the blood flow to avoid bleeding. The upper ligature is necessary for fixing the catheter in the artery during the operation. Note that the tissues must be protected from drying by constantly adding saline (around 37 °C) into the surgery area to keep the tissue elastic and minimize the risk of renal artery damage.

A catheter consisting of two tubes of different diameters connected to each other (Figure 1I) was used in the procedure. The thin part of the catheter (5 mm, PU tubing, 32ga/0.8Fr, 0.005 × 0.010 in, Instech Laboratories, Inc.,Plymouth Meeting, PA, USA) was completely implanted into the femoral artery (Figure 1D) through a small puncture. Note that the thin catheter tip should be beveled for easier implantation into the femoral artery. The thick part of the catheter (70 mm, PE-10, Scientific Commodities INC., Lake Havasu City, AZ, USA) serves as an adapter and allows injection of solutions and suspensions into the catheter with a syringe with a 30 G needle. Note that prior to implantation, the catheter should be filled with saline to avoid air vessel embolization. During the catheter implantation, the artery should be very taut. The catheter should not be strongly squeezed with forceps and stretched to avoid its deformation and loss of mechanical properties. During implantation, it is necessary to periodically check the catheter localization in the artery by pulling the syringe plunger. If blood enters the catheter easily (as shown in Figure 1D), this indicates that the catheter is inside an artery. If blood does not enter the catheter, this indicates that it has been mistakenly implanted in the femoral vein or that the catheter has punctured the artery wall and is located in the surrounding tissue. If the thin part of the catheter cannot be fully implanted and the catheter is stuck in the artery by about half (2.0–2.5 cm), it is necessary to change the mouse hind paws’ position and make themtaut along the tail. As a rule, it becomes much easier to push the catheter into the aorta after such manipulation.

At the second stage of the operation, the animal is turned over, as shown in Figure 1E. A roller (d = 1 cm) is put under the mouse body (Figure 1E). In this case, the kidney is clearly visible through the skin. To access the renal artery, a small skin and muscle incision (1 cm) was made over the kidney or slightly closer to the spine. Next, a large renal vein should be found with the tweezers, after which the tissues were cleared using a soft cotton swab to better visualize the junction between the aorta and the renal artery. The artery tightly adjoins the renal vein as a rule, due to it being necessary to exclude vein damage during this manipulation. Next, the catheter should be slowly pulled out of the femoral artery while constantly “palpating” it inside the aorta with forceps. When the thin catheter tip is located 2–3 mm above the junction between the aorta and the renal artery, it can be easily transferred from the aorta to the renal artery using two forceps (Figure 1F). Note that in this case, blood continues to flow into the kidney from the renal artery to some extent because the renal artery is approximately 0.35–0.55 mm in diameter [45], which is slightly larger than the diameter of a thin catheter (0.25 mm).

To increase the efficiency of a solution or suspension targeting the kidney, the catheter must be tightly pressed by the artery walls at the time of insertion. This allows keeping the catheter in the renal artery during injection and minimizes the ingress of the injected solution into the aorta. In this case, the target kidney first turns darker in color due to the blockage of blood flow (Figure 1G). Then, on the contrary, it becomes light due to the replacement of blood in the kidney vessels by the injected solution or suspension (Figure 1H). All these factors are reliable evidence of the manipulation’s correctness. In some cases, it is not possible to unambiguously see changes in the kidney color during injection of the solution, even with a correctly performed operation and reliably registered catheter localization in the renal artery. This may be due to anomalies in the structure of the kidneys vessels and the presence of small accessory renal arteries [51]. It is worth avoiding deeper penetration of the catheter into the renal artery due to the presence of a bifurcation, because in this case, the injected solution or suspension can only reach one half of the kidney. Although, the described strategy (injection through one of the two branches of the renal artery) may be advantageous, for instance, in cases of localized cancer therapy.

The moment when the container suspension has already been injected, but the tweezers are still blocking the blood flow, is the most promising for external stimulation of the containers. It may be useful to externally stimulate the release of the encapsulated drug [52] or to attract magnetically sensitive containers to the vessel walls [28] in order to make retention more effective. This moment may also be the most successful for the implementation of local photodynamic therapy [53,54] or magnetic hyperthermia [55]. This is due to the fact that at this moment, most of the drug dosage is localized in the vessels of the target kidney with the optimal selection of the injection volume. In clinical practice, standard endovascular catheters with balloons can be used to block blood flow temporarily [13]. In the experiments, the blood flow was not blocked for more than 3–5 min, which allowed preserving the normal morphological state of the target kidney tissues (Figure 1J). Nonetheless, the blood flow blocking time can be increased at the request of the experimenter. However, it should be taken into account that a prolonged blockage of blood flow may lead to a failure to restore blood supply in the future [15,56]. It is also worth avoiding a strong and prolonged mechanical exposure on the vessels even before the catheter implantation into the artery. The kidney should retain its normal coloration and blood supply during all stages of the operation up to this point. Only in this case is it possible to correctly assess the effects of injected drugs and drug containers on the renal tissue.

In the last stage of the operation, the catheter is completely pulled out of the vessels and the upper ligature is tightened. Note that, when the operation is carried out quickly (30–40 min), the hind paws’ blood supply does not suffer due to the femoral artery blocking. This is explained by the presence of anastomoses between the vessels in the hind paw muscles [57] and collateral artery enlargement [58]. In any case, it is worth injecting a heparin solution (10 IU through the tail vein (maximum 5 mL/kg)) after suturing the muscle and skin as a prophylaxis for extremity thrombosis as a result of endovascular intervention [59,60]. The proposed method does not imply disruption (puncture) of the renal artery or aorta and is suitable for long-term chronic experiments on mice.

### 3.2. Reversible/Irreversible Changes of Kidney Blood Flow Associated with Various Sizes and Concentrations of Microcapsules Injected via Renal Artery

**Figure 2:**
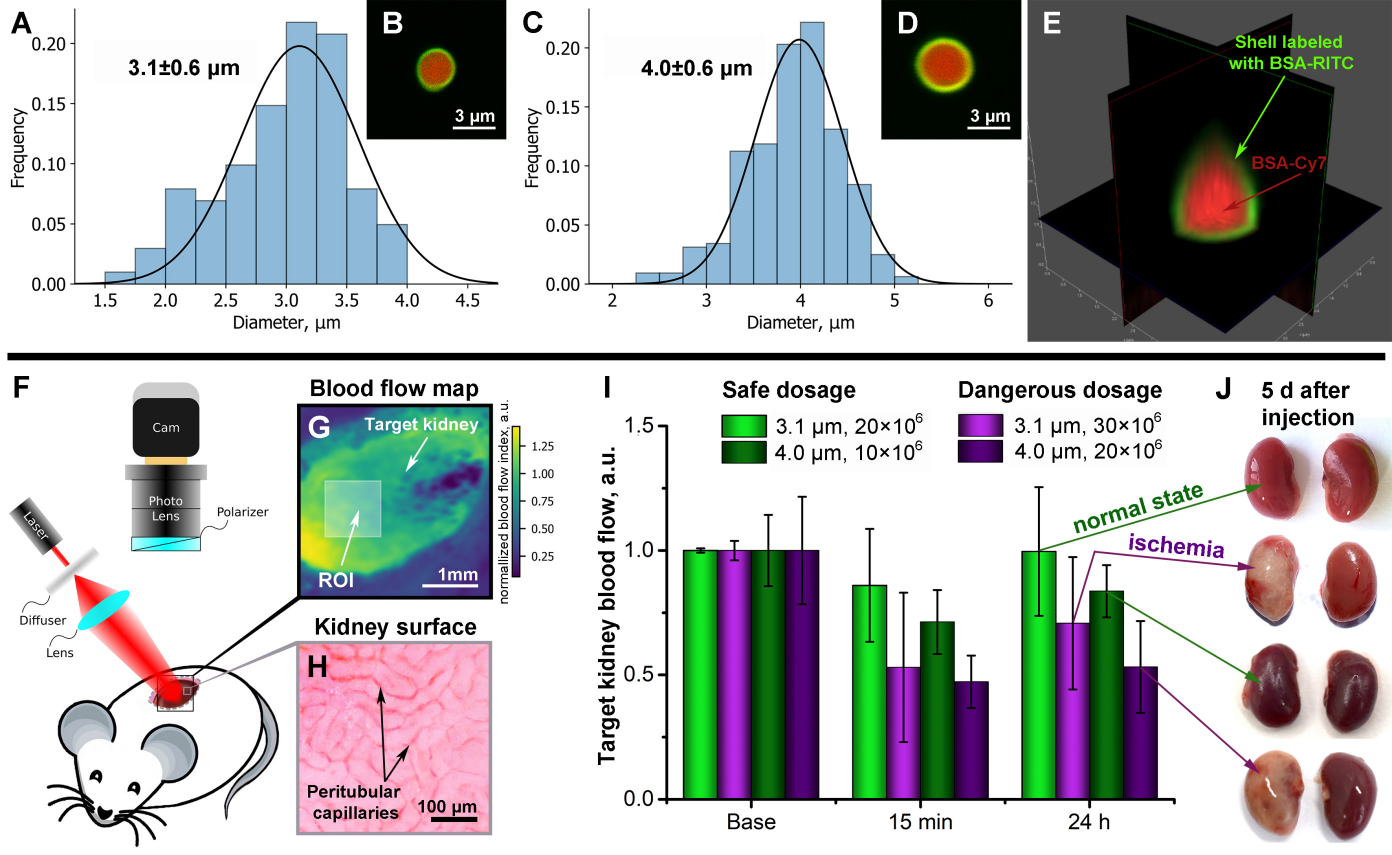
Characterization of polyelectrolyte microcapsules with two sizes (**A**–**D**). Size distribution with Gaussian fit (**A**,**C**) and typical CLSM images (**B**,**D**) in aqueous solution. CLSM 3D image of typical polyelectrolyte microcapsule (**E**). Green color indicates a shell labeled with BSA-RITC. Red color indicates the encapsulated cargo (BSA conjugated with Cy7) (**B**,**D**,**E**). The general scheme of the experiment on the registration of mouse kidney blood flow using the laser speckle contrast analysis system (**F**). Typical laser speckle contrast image (LSCI) of the target kidney surface ((**G**); scale bar, 1 mm). Bright-field microscopy image of superficial peritubular capillaries of the mouse’s kidney that forms the LSCI signal (**H**). Dose-dependent changes of target kidney blood flow before and 15 min and 24 h after the different size microcapsules’ injection via the left renal artery measured using the laser speckle contrast analysis system (**I**). The typical photos of the target kidney 5 days after microcapsules’ injection via the renal artery (**J**).

Our previous studies using a laser speckle imaging system [22,25] and optical coherence tomography [29] showed the importance of monitoring the blood flow of the target organ for choosing a safe dosage of drug containers. In this regard, in the second stage of the study, the mutual influence of the container’s size and concentration on the reversibility/irreversibility of changes in the kidney blood flow was studied. Microcapsules based on polyarginine and dextran sulfate were synthesized in two sizes 3.1 ± 0.6 μm and 4.0 ± 0.6 μm (Figure 2A–D). The shell of the capsules was labeled with BSA-RITC. BSA-Cy7 conjugate as a model of macromolecular cargo was loaded into the inner space (Figure 2B,D,E). Capsules containing both fluorescent dyes Cy7 (in the core) and RITC (in the shell) were used at all stages of the study. A representative image of such a capsule is shown in Figure 2E. Microcapsules based on polyarginine and dextran sulfate have a number of unique properties such as (i) the ability to mechanically deform due to the absence of a hard core, which could provide normal blood flow in the vessels where the capsules accumulated [28,29], (ii) biocompatibility and very low toxicity [30,31], (iii) high stability [27], (iv) complete biodegradation within a few weeks [32], and (v) the ability to be further modified with fluorescent agents [33,34] and nanoparticles [35,36]. However, encapsulation of low-molecular-weight therapeutic agents is still challenging.

Figure 2F shows the general scheme of the experiment on registration of kidney blood flow in mice using a laser speckle contrast analysis system (LSCI). To record blood flow, a small incision in the skin and muscles was made above the target kidney under general anesthesia, which allowed obtaining a laser speckle contrast signal (Figure 2G) from the superficial peritubular capillaries of the mouse’s kidney (Figure 2H).

The histograms in Figure 2I and Figure S2 (see Supplementary Materials) clearly demonstrate that any manipulations performed have an effect on the kidneys’ blood flow. However, in the case of capsules with a smaller size (3.1 ± 0.6 μm) at a dosage of 20 × 10${}^{6}$ (Figure 2I) or saline injection (Figure S2), the decrease in blood flow at the first min was the lowest and did not exceed 20%, and then, there was a recovery by 24 h. In contrast, the administration of the same dose (20 × 10${}^{6}$) of capsules with a bigger size (4.0 ± 0.6 μm) led to irreversible changes (the drop was 53% and 47% at 15 min and 24 h after administration, respectively). The blood flow’s non-restoration led to ischemic changes in the target kidney tissue, which were clearly visible by Day 5 of the experiment (Figure 2J). It is worth noting that small changes in blood flow even in the control group of animals were observed (Figure S2). Thus, one can conclude that blood flow changes by no more than 20% are a physiologically normal phenomenon that can be caused by both external factors and natural processes. In this regard, the dosages 20 × 10${}^{6}$ and 10 × 10${}^{6}$ for 3.1 ± 0.6 μm- and 4.0 ± 0.6 μm-sized capsules, respectively, were found relatively safe for injection into the mouse renal artery in a volume of 10 μL.

An important result of this stage of research is the establishment of the relationship between the dose and/or size of the administered carriers and the effect on the renal blood flow. For each type of carrier, the safe dose will vary. The size of the injected carriers, their rigidity, and the chemical composition of the outer shell, which primarily interacts with the endothelium, will have a huge impact. Thus, the larger the size, the smaller the amount the can be considered safe, or the more rigid the media used, the smaller the amount is to be safe, and so on. Obviously, this topic needs a preliminary study and will be of great importance for both fundamental and applied science.

### 3.3. Optoacoustic Imaging of Blood Circulation in the Target Kidney after the Microcapsules’ Administration

The data from laser speckle contrast analysis (Figure 2I) showed the most pronounced changes in blood flow for larger-sized capsules (4.0 ± 0.6 μm). Thus, the effect of such capsules on the functional state of the kidney vessels and the tissues’ morphological condition were studied in more detail in order to confirm their safety.

**Figure 3:**
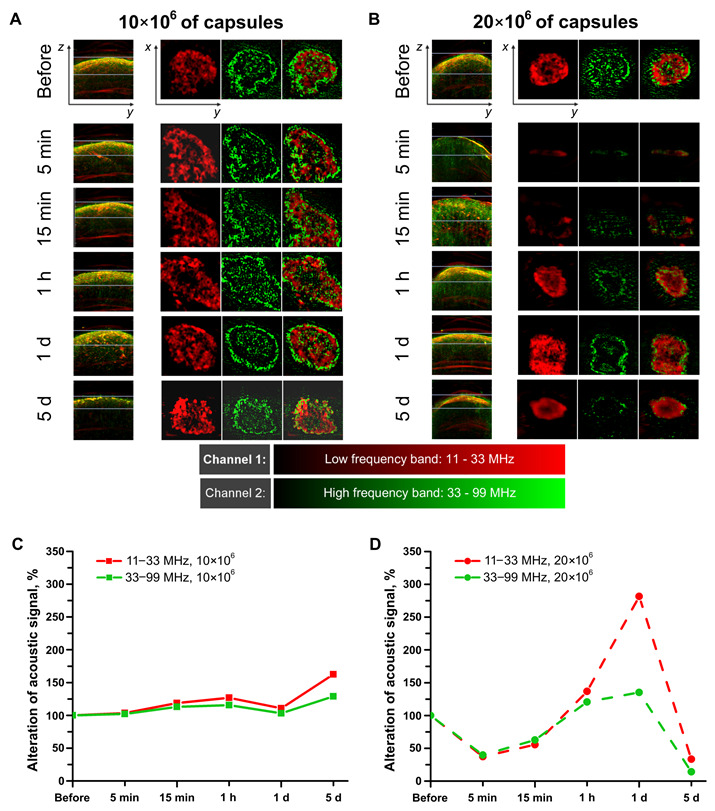
Optoacoustic imaging of the kidney’s vascular bed. Observation of blood circulation in the target kidney after 10 × 10${}^{6}$ (**A**) and 20 × 10${}^{6}$ (**B**) capsules’ injection. The side of the square for the ZY projection is 4 mm and for the XY projection is 2 mm. Alteration of the acoustic signal for the low- and high-frequency range in the target kidney after 10 × 10${}^{6}$ (**C**) and 20 × 10${}^{6}$ (**D**) capsules’ injection.

The dynamics of the blood circulation in the kidneys before and after the capsules’ administration at two dosages (10 × 10${}^{6}$ and 20 × 10${}^{6}$) was evaluated via a raster scanning optoacoustic mesoscope (RSOM) for quick visual identification of the harmful impact of unsafe dosage besides deeper signal detection compared to LSCI. These results are shown on Figure 3A,B. As is known, the main signal source in a living organism during photoacoustic imaging at 532 nm is hemoglobin [61,62], carried by erythrocytes in blood vessels. Optoacoustic imaging at low frequencies (20–30 MHz) had a resolution of about 70–100 μm [63]. Larger vessels have an acoustic signal in the range of 11–33 MHz, in contrast to small capillaries with a high-frequency range 33–99 MHz.

Concerning Figure 3A,B, it can be noted that before the capsules’ injection, both ranges are well visualized: the “red band” (large vessels) is detected more clearly in the near-surface region, while the “green band” (small capillaries) has a more uniform distribution of the signal over the depth. Figure S3 (see Supplementary Materials) clearly illustrates how the pattern of the detected acoustic signal changes depending on the depth of the vessels and the capillaries’ location in the kidney. Thus, the signal from large vessels is not detected in the center of the section under consideration as one moves along the Z axis (the direction from the upper surface of the kidney placed directly under the transducer to the area located inside the body). Single large vessels (11–33 MHz) are visualized no deeper than 0.75 mm, and as they reach the surface of the kidney, they form clots, which may be caused by the presence of glomeruli in the cortical region or could be a feature of this imaging method due to the overlaying of the signal from vessels located in nearby layers. The high-frequency signal (33–99 MHz) from small vessels and capillaries is determined throughout the depth of the kidney.

As can be seen from Figure 3A, the capsules’ administration at a dose of 10 × 10${}^{6}$ did not lead to changes in the acoustic signal in both ranges. This visual observation was confirmed by quantitative data. In the rLabs software, the average values of the acoustic signal intensity were obtained for each range separately in the areas selected on the ZY projections. The percentages of the intensity of acoustic signals relative to their values before the injection were calculated. Therefore, Figure 3C shows that there were no dramatic changes in the detected signals. However, in the case of an unsafe dose (20 × 10${}^{6}$ capsules), both very strong visual and quantitative changes occurred (Figure 3B,D). Then, 5 and 15 min after the injection, the acoustic signal in both ranges almost completely disappeared, which may indicate severe circulatory disorders in this organ. There was some recovery at the hour point. The image with a low-frequency signal looks much better as the pattern goes up to the original view, while high frequencies are detected only in certain areas close to the surface of the kidney. One day after the injection of capsules (20 × 10${}^{6}$), the low-frequency signal had an abnormally high intensity, although high frequencies were still determined only in certain areas and did not fill the entire volume of the organ, as before the injections. By Day 5 of the experiment, the high-frequency signal weakened even more, rather than recovering. The intensity of the low-frequency signal also fell, and the visual representation of this signal underwent strong changes. The picture shows only a blurred red spot without a characteristic pattern, which is present on the images before injection or when a safe dose is administered.

The experiments performed showed that RSOM can be successfully used to monitor acute and chronic pathological changes in the renal vasculature. Previous studies have shown imaging of large renal vessels (renal arteries and veins) using a planar PA scanner based on a Fabry–Perot polymer film ultrasound sensor [14,64]. This research method makes it possible to non-invasively observe changes in the blood supply to the kidneys, but does not make it possible to see changes in blood flow in small vessels penetrating the entire organ. However, with the help of RSOM, we were able to observe changes in vessels and capillaries located in the cortical layer and deeper.

### 3.4. Capsules’ Localization and following Elimination from Target Kidney

**Figure 4:**
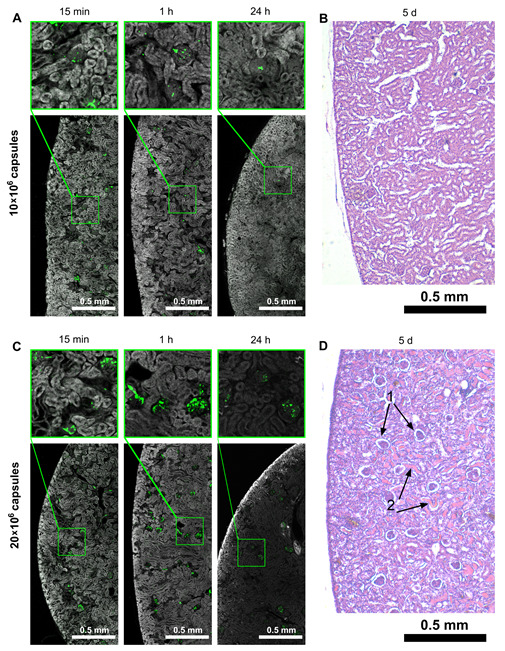
Distribution of capsules administered to the left kidney and dose-dependent impact on the kidney’s structure. Fluorescent microscopy of the target kidney’s cryosections 15 min, 1, and 24 h after microcapsules’ injection at a dose of 10 × 10${}^{6}$ (**A**). Histological section of the target kidney 5 days after 10 × 10${}^{6}$ capsules’ administration (H & E staining) (**B**). Fluorescent microscopy of the target kidney’s cryosections 15 min, 1, and 24 h after microcapsules’ injection at a dose of 20 × 10${}^{6}$ (**C**). Histological section of the target kidney 5 days after 20 × 10${}^{6}$ capsules’ administration (H & E staining). 1—Bowman’s capsule is dilated, 2—plasma proteins in the lumen of the tubule (**D**).

Cryosections of the target kidney were imaged using a confocal laser scanning microscope (Leica TCS SP8 X) (Figure 4A,C) to study both the distribution of capsules after injection and the subsequent dynamics of their elimination. Fluorescence was provided by the BSA-RITC conjugate (green) and autofluorescence (gray). Thus, thanks to natural fluorescence, it was possible to clearly show the structure of the kidney (glomeruli and renal tubules) (see the Supplementary Materials, Figure S4).

The fluorescence microscopy images (Figure 4A,C) give insight into three trends. Firstly, as shown earlier in [22,29], the capsules get stuck in the glomeruli (images corresponding to 15 min after administration). The larger the dose of the capsules, the more glomeruli are filled with them. Secondly, in the case of safe dose administration, the number of capsules gradually decreases, and single objects remain in the field of view in the target organ by 24 h. On the other hand, there is no significant reduction in the number of capsules even 24 h after the injection of an unsafe dose. The glomeruli are still filled with capsules. This result could be regarded positively from the point of view of prolonging the presence of the drug delivery system in the target organ, if not for pathological changes, which are noticeable by a sharp drop in autofluorescence one day after administration. This is the third trend.

There are many works where the physiological state of the object under study is assessed by measuring the autofluorescence of cells and biological tissues [65]. Native fluorescence in cells is provided by endogenous biomolecules capable of light emission when excited at a suitable wavelength. Such endogenous fluorophores include proteins, porphyrins, coenzyme NAD(P)H, vitamins, fatty acids, flavins, and lipofuscin-like lipopigment [66]. Changes in fluorescence intensity or shifts in the spectra of these biomolecules are associated with certain, often pathological processes, which makes endogenous fluorophores excellent biomarkers [67].

Figure 4C clearly shows a drop in autofluorescence intensity 24 h after administration of an unsafe dose, indicating ischemia. Thus, in the works [68,69], a significant decrease in the autofluorescence of biological tissues of the rat kidney was shown after stopping the blood supply to this organ. Hsueh-Han Lu et al. [70] hypothesized that a decrease in molecular oxygen (O_2_) entering the cells during ischemia leads to an increase in the amount of reduced flavin adenine dinucleotide (FADH2) compared to its oxidized form (FAD). The authors of this work showed that the main contribution to cell autofluorescence excited by 445 nm is made by flavins in mitochondria and determined the oxidized form of FAD as fluorescent and the reduced form (FADH2) as non-fluorescent. However, this is not entirely accurate. In [71], changes in the spectra were shown during the transition from FAD to FADH2. The absorption maximum for FAD is at 450 nm, while for FADH2, the maximum shifts to 345 nm, and after 400 nm, the absorption is almost zero, which leads to a sharp drop in fluorescence upon excitation at a frequency above 400 nm. Thus, the transition from bright to weak autofluorescence in Figure 4C indicates a lack of oxygen reaching the cells of the target kidney 24 h after capsule administration and is a marker of ischemia when an unsafe dose of capsules (20 × 10${}^{6}$) has been injected.

Figure 4B shows that the targeted kidney tissue on Day 5 after the 10 × 10${}^{6}$ microcapsules’ injection has a normal morphological structure, unlike the targeted kidney with 20 × 10${}^{6}$ microcapsules, which looks edematous (Figure 4D). The targeted kidney with a high dosage is diffusely infiltrated with leukocyte cells, which may indicate the onset of generalized inflammation, namely nephritis. Nephrocytes of the kidney tubules have fuzzy contours; the nuclei are enlarged; the cells have a pronounced vacuolization. The lumen of the tubules is narrowed due to the edema of nephrocytes or is disproportionately expanded. The substance that perceives eosin is clearly visible in the lumen of some tubules, which may indicate the perspiration of a large amount of plasma proteins through the damaged capillaries of the glomeruli. Bowman’s capsule is dilated compared to that of the glomeruli of a kidney with 10 × 10${}^{6}$ capsules (Figure 4B). The capillary of glomeruli has different sizes, and glomerular endothelial cells are enlarged. The glomeruli respond well to hematoxylin, probably due to acidosis caused by ischemia. These changes are characteristic of the early stages of tubulointerstitial nephritis with some damage to the glomeruli caused by arterial occlusion [72,73].

### 3.5. Biodistribution Kinetics of the Fluorescent Marker after Administration to the Target Kidney in Free and Encapsulated Form

After establishing a safe dose and confirming the localization of injected capsules in the structural elements of the target kidney, it is reasonable to consider the distribution of the fluorescent conjugate (BSA-Cy7) encapsulated in the inner space throughout the body immediately after administration and its further migration trajectory. For this purpose, a comparative study of the fluorescent signal presence in organs (kidneys, lungs, heart, liver, spleen, stomach, intestines, appendix) was carried out after left renal artery administration of a fluorescent conjugate in the composition of polymer capsules and free form. The migration dynamics of the fluorescent signal source was monitored using a fluorescent tomograph, IVIS Lumina III (PerkinElmer).

**Figure 5:**
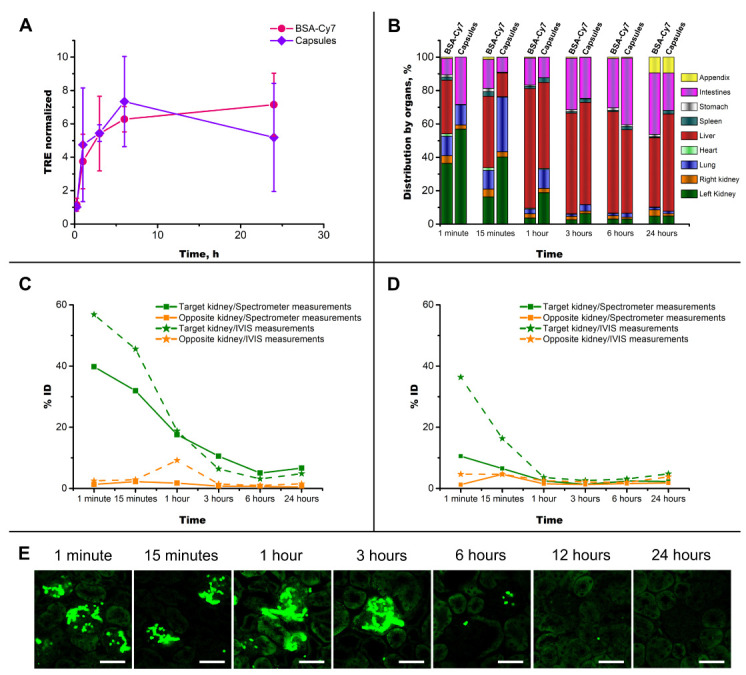
Biodistribution of fluorescent dye (Cy7) after intra-arterial injection of capsules labeled with BSA-Cy7 and its free solution (**A**–**D**). Time dependence of integrated total radiant efficiency by all dissected organs (**A**). Kinetics of the biodistribution of Cy7 (**B**). Time dependence of Cy7 content in the kidneys expressed as a percentage of injected dose after administration of capsules labeled with BSA-Cy7 (**C**) and free BSA-Cy7 conjugate (**D**). Fluorescent microscopy of the kidney’s cryosections after 10 × 10${}^{6}$ capsules’ injection (**E**). The detection channel was adjusted to RITC fluorescence. The scale bar is 50 μm.

The first important result of observing the change in the fluorescent signal is its overall increase over time. Therefore, on the graph in Figure 5A, which characterizes the total fluorescent signal (total radiant efficiency) for all the dissected organs, there is a tendency to increase regardless of the administered object (free or encapsulated fluorescent conjugate), which is similar to the results described in [74]. A significant change in intensity may be associated with the active interaction of the Cy7 dye with biomolecules, such as proteins, since it is known from a number of works that the higher the protein concentration, the higher the fluorescence intensity of cyanine dyes is [75,76,77]. Thus, for the case of capsule administration, this indirectly indicates their rapid degradation and release of the BSA-Cy7 conjugate encapsulated in the inner space.

A detailed consideration of the kinetics of the redistribution of the fluorescent signal throughout the body reveals several important features. Firstly, the capsules’ administration through the renal artery provided a significantly greater fluorescence in the target kidney compared to the opposite during 24 h of observation, while 3 h after the free conjugate injection, the fluorescent signal in both kidneys became the equal. Several papers presented the results of an effective increase in the concentration of intravenously administered delivery systems based on polypeptides [78,79], nanoparticles [80,81], triblock amphiphilic polymers [82], micelles [83], and macrophage microvesicles [84]. However, the administered carriers are distributed equally in the both kidneys, which may not be desirable if only one kidney needs therapy.

Secondly, the injection of a fluorescent conjugate in encapsulated form ensures its longer retention in the target kidney. Thus, a semi-quantitative determination of the injected dose proportion in different organs shows that after intra-arterial administration of capsules, up to six-times more fluorescent agent remained in the left kidney compared to its administration in the free form (the maximum difference was noted 1 h after administration).

Thirdly, of particular interest is the dynamics of fluorescence in the liver. Thus, the liver remains free of Cy7 fluorescence 1 min after the capsules’ administration. Even after 15 min, the portion of fluorescence in the liver remained almost three-times less than the portion of fluorescence distributed in the target kidney. The intensity of fluorescence in the liver increased and reached 60% of the total fluorescence of all organs over time. However, this fluorescent signal distribution, which is not typical for the injection of micron-sized drug delivery systems, indicates the avoidance of the well-known “first pass effect” [85] specific to intravenous injection. It should also be noted that the increase in the fluorescent signal in the liver can be associated not only directly with the migration of capsules, but also with their degradation and the interaction of Cy7 with the micro-environment in the liver, as noted earlier. It is not possible to separate these two processes at this stage of research.

The calculation of the fluorescent marker distribution based on IVIS measurements is very rough due to many assumptions (for instance, consideration of a limited number of organs, possible losses during photon detection from organs’ depth, low resolution). A comparison was made of the percentage of injected dose (%ID) values obtained from image analysis using the Living Image®PerkinElmer software and using a spectrophotometric assessment of the Cy7 content in a specific organ after its homogenization (the homogenization protocol was described in [22]). As shown in the graphs (Figure 5C,D), the trends for both methods of calculating %ID were the same. However, the values of %ID at the first min after administration, obtained using IVIS, were strongly overestimated, which, we believe, was due to the consideration of a certain set of organs and the exclusion of the part of the administered dose that still circulated throughout the rest of the body from measurements and subsequent calculations.

The study of the capsules’ shell degradation while in the kidney is of great importance due to the envisaged clinical applications. For this purpose, a fluorescent conjugate BSA-RITC was incorporated into the shell structure, which made it possible to separate the signal from the substance loaded inside the capsules and located in the shell during fluorescence microscopy. By comparing the graph (Figure 5C) with the fluorescence image line (Figure 5E) of the target kidney cortex after the intra-arterial injection of capsules, the following processes can be observed.

The fluorescent image corresponding to the first minute after injection exhibited an accumulation of microcapsules in tortuous glomerular vessels and staining of surrounding tissues with released fluorescent dye (RITC). The release rate of the substance loaded into the microcapsules at the first moment was much higher than later on during in vitro incubation in 0.15 M NaCl at 37 °C [86]. Thus, in the first micrograph, the effect of the first “burst release” can be observed when the microcapsules enter the blood. In the case of normal blood circulation in the organ, the incoming blood flushes out the free fluorescent agent after 15 min, leaving capsule clots in the glomerular vessels. The value of %ID (Figure 5C) also showed a slight decrease in the amount of fluorescent marker in the left kidney.

There were still many capsules in the glomeruli 1 and 3 h after administration, but according to the staining of the surrounding tissues, there was an active degradation of the capsule shell (please note that the fluorescence images in Figure 5E show a detection channel tuned to the fluorescent dye RITC, which was localized exactly in the capsule shell; see the Materials and Methods: Microcapsules’ Preparation). The molecules of the fluorescent dye leaving the capsule shell partly diffused into surrounding tissue or washed out with the blood stream, which corresponds to a steady decrease in in %ID in Figure 5C. Only several microcapsules remained in the glomeruli at the 6 and 12 h time points, but the soaking of the tissue by RITC was preserved.

The glomeruli were completely decapsulated 1 day after injection, but overall tissue fluorescence was still present, consistent with a 12 h %ID value (Figure 5C). However, it should be noted that the fluorescent molecules that come to the kidney for a further excretion from the body will also contribute to the overall signal.

## 4. Discussion

All the experiments carried out allowed building a chronology of events according to two scenarios. Firstly, with the correct implementation of all surgical procedures, renal artery catheterization did not interfere with the blood supply to the target kidney and allowed injection of the dosage form (biologically active or therapeutic substance loaded into polymeric microcapsules) directly into the organ being treated (Figure 1). After injection, the microcapsules were deposited in the vasculature of the target kidney. Short-term (1 min) restriction of the blood supply to the organ allowed some of the capsules to attach to the endothelial wall. With the restoration of blood supply, the process of cleansing the kidney of capsules loosely adhering to the endothelium began (Figure 5B). The straight-line sections of the vessels were largely free from the injected carriers, while the tortuous vessels of the glomeruli retained a large number of capsules (Figure 4A,C). However, the accumulation of capsules in the glomeruli at a rationally selected dose did not lead to severe disturbances in the overall blood circulation in the organ (Figure 2I and Figure 3A,C). This way of administration resulted in the accumulation of more delivered cargo in the target kidney compared to free substance administration for an extended period of time (up to 6 h) (Figure 5B). The opposite kidney could remain free from the treatment burden (Figure 5B–D) due to the proposed method of administration. Capsules located in the vessels of the kidney underwent both washing out with the bloodstream and gradual degradation of the membrane, followed by soaking of the surrounding tissues with molecules that had left the capsules (Figure 5E). The vessels were almost completely cleared of the injected capsules 12 h after the injection (Figure 4A and Figure 5E), and on Day 5 after injection, no macroscopic nor microscopic pathological changes in the kidney tissues were observed (Figure 2J and Figure 4B).

The second scenario was performed with an excessive amount of injected carriers and led to an irreversible change in blood circulation in the kidney. After opening the blood supply to the target organ, the injected capsules filled the glomerular vessels so tightly, that blood could not pass through and ischemia occurred (Figure 4C,D). The drop in blood flow detected by laser speckle contrast and optoacoustic imaging was very significant and did not recover over time (Figure 2I and Figure 3B,D). Ischemic damage was visible at both the micro and macro levels at 5 days post-injection (Figure 2J and Figure 4D). Thus, it is important to pay attention not only to the development of drug delivery systems, but also to their rational use.

## 5. Conclusions

The need to localize active substances as much as possible in renal therapy has been noted in many works [78,87]. A novel effective approach to drug delivery based on a combination of endovascular surgery and the use of polymeric microcapsules was reported. Each of the mentioned approaches has strengths and weaknesses. In the present study, various parameters of both the surgical procedure and the size/dosage of microcapsules were considered. All characteristics were determined to achieve the strongest effect of localization and retention of a model macromolecular substance without leading to pathological processes developing. The detailed description of the surgical procedure for mouse renal artery catheterization provided in the article explained step by step how and why this protocol should be followed. A comparative analysis of the effect on blood flow of different doses of capsules with various sizes showed that the larger the size of the administered object, the lower the dose must be to be considered safe. An increase in microcapsules’ size by 29% (from 3.1 to 4.0 μm) requires a dose reduction of at least 50%. However, at the same time, we emphasize that moderate blood flow disturbances caused by the injection of micron-sized objects can be reversible and not lead to pathological changes, since a decrease in blood flow by 20% can occur in the kidney without any manipulation, as shown here. It is important to rationally choose the dose of injection. Thanks to optoacoustic imaging of the vascular bed of the kidney, it was shown that with an unsafe dose of capsules, blood circulation decreased both in large vessels and in capillaries. Such disorders appeared immediately after the injection, and recovery did not occur even at Day 5 after the injection. In contrast, when a safe dose was administered, significant changes in blood flow were not observed. It is also proposed to consider RSOM imaging as a method for diagnosing renal vascular pathologies. Observation of the redistribution kinetics of the fluorescent agent in the body after intra-arterial injection of encapsulated and free BSA-Cy7 into the mouse kidney made it possible to demonstrate the efficiency of the localization and retention of the loaded substance in the target kidney upon microcapsules’ administration. The proposed approach to the treatment of kidney diseases is promising and requires further research on the effectiveness of its use for curing various renal pathologies, including cancer and chronic diseases. The described technique will be extremely useful in oncotherapy, as it can help not only reduce the systemic side effects on other organs, but also minimize the drug influence on a healthy contralateral kidney. The described approach could be effective for targeting a wide range of drug delivery systems. In the context of the usage the layer-by-layer microcapsules, it will be relevant for the delivery of high-molecular cargoes such as proteins, peptides, ferments, and genetic material.

## Data Availability

The data presented in this study are available upon request from the corresponding author. The data are not publicly available due to several reasons: large size, overall complexity of processing protocols, storage in proprietary formats.

## Supplementary Materials

The following are available online at https://www.mdpi.com/article/10.3390/pharmaceutics14051056/s1, Figure S1: Optoacoustic imaging. Photograph of the optoacoustic visualization process (A). Pre-imaging of the left kidney shows the clear border of the organ (B), Figure S2: Changes of target kidney blood flow before and 15 min and 24 h after saline injection via the left renal artery (sham operation) and without any manipulation (control) measured by the laser speckle contrast analysis system, Figure S3: Optoacoustic imaging of the left kidney before capsules’ injection at 2 planes that correspond to the cortex layer (1st plane) and a deeper layer where the medulla region is present (2nd plane) Optoacoustic imaging of the left kidney before capsules injection at 2 planes that correspond to cortex layer (1st plane) and deeper layer where present medulla region (2nd plane). Optoacoustic imaging of large and small vessels in the left kidney (A). Histological image of the left kidney without any injection. Thickness of histological samples is 5 μm; dyes: hematoxylin and eosin (B), Figure S4: Fluorescent microscopy of the kidney’s cryosections. Autofluorescence channel (405 nm excitation, 415–470 nm emission detection). Red arrows point to glomeruli; blue arrows point to renal tubules.
