# Supplementary material for: Renal Artery Catheterization for Microcapsules’ Targeted Delivery to the Mouse Kidney
Source: Pharmaceutics. 2022 May 14;14(5):1056. doi: 10.3390/pharmaceutics14051056 (PMC9144148; doi:10.3390/pharmaceutics14051056)
Supplement: Supplementary file 1 [file pharmaceutics-14-01056-s001.zip › pharmaceutics-1708693-supplementary.pdf]

Supplementary Materials: Renal Artery Catheterization for Microcapsules Targeted Delivery to the Mouse Kidney

Olga I. Gusliakova [1,*], Ekaterina S. Prikhozhdenko [1], Valentina O. Plastun [1], Oksana A. Mayorova [1], Natalia A. Shushunova [1], Arkady S. Abdurashitov [2], Oleg A. Kulikov [3], Maxim A. Abakumov [4], Dmitry A. Gorin [5], Gleb B. Sukhorukov [2,6] and Olga A. Sindeeva [1,2,*]

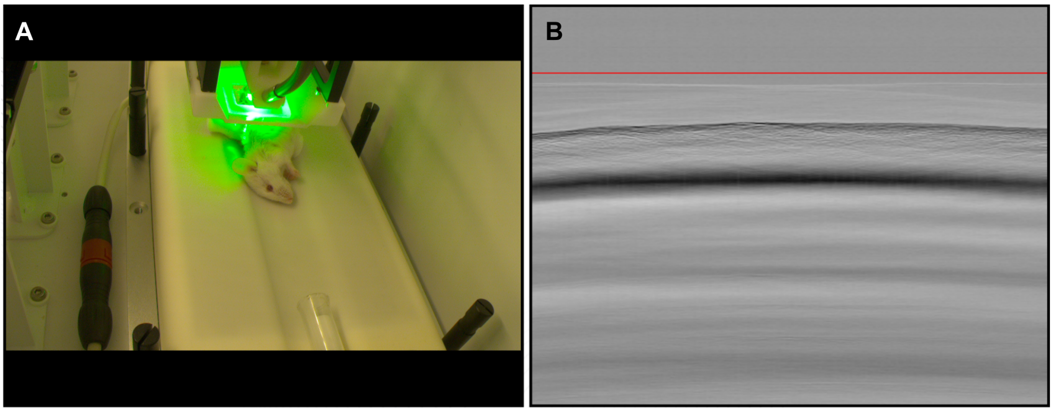

Figure S1. Optoacoustic imaging. Photograph of optoacoustic visualization process (A). Pre-Imaging of the left kidney shows the clear border of organ (B).

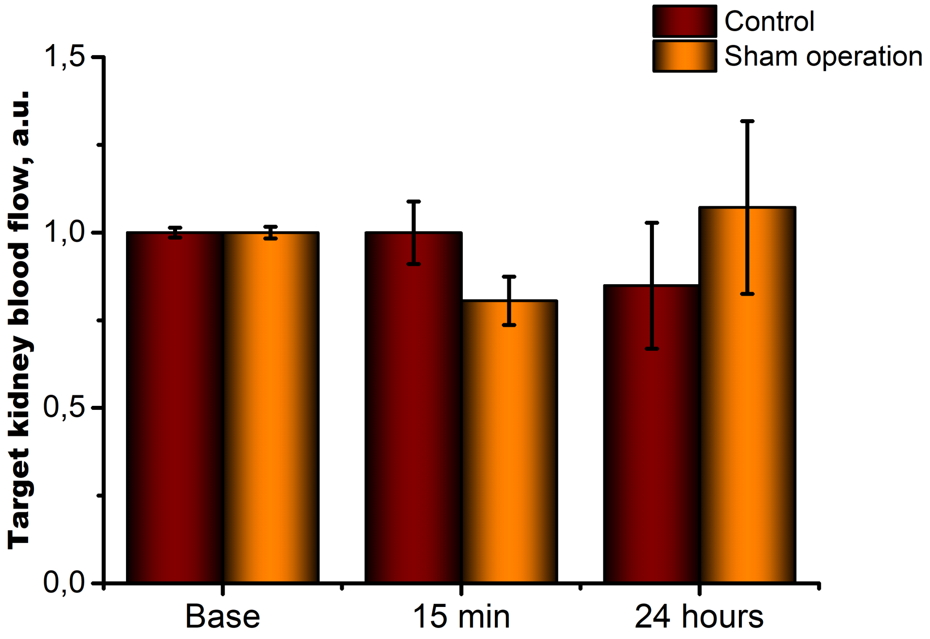

Figure S2. Changes of target kidney blood flow before and 15 min, 24 h after saline injection via left renal artery (sham operation) and without any manipulation (control) measured by laser speckle contrast analysis system.

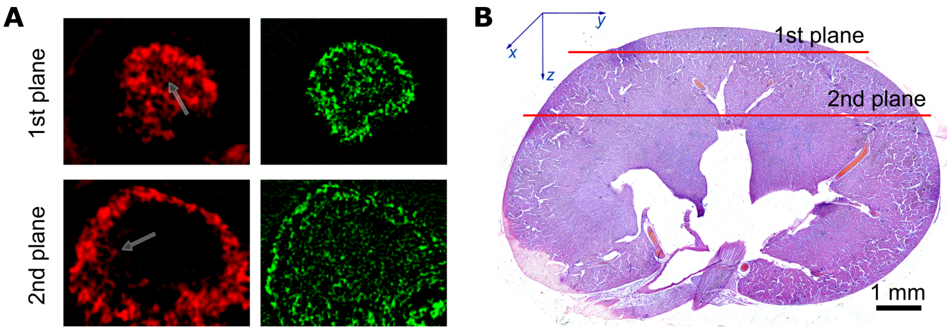

Figure S3. Optoacoustic imaging of the left kidney before capsules injection at 2 planes that correspond to cortex layer (1st plane) and deeper layer where present medulla region (2nd plane). Optoacoustic imaging of large and small vessels in the left kidney (A). Histological image of the left kidney without any injection. Thickness of histological samples is 5 µm; dyes: hematoxylin and eosin (B).

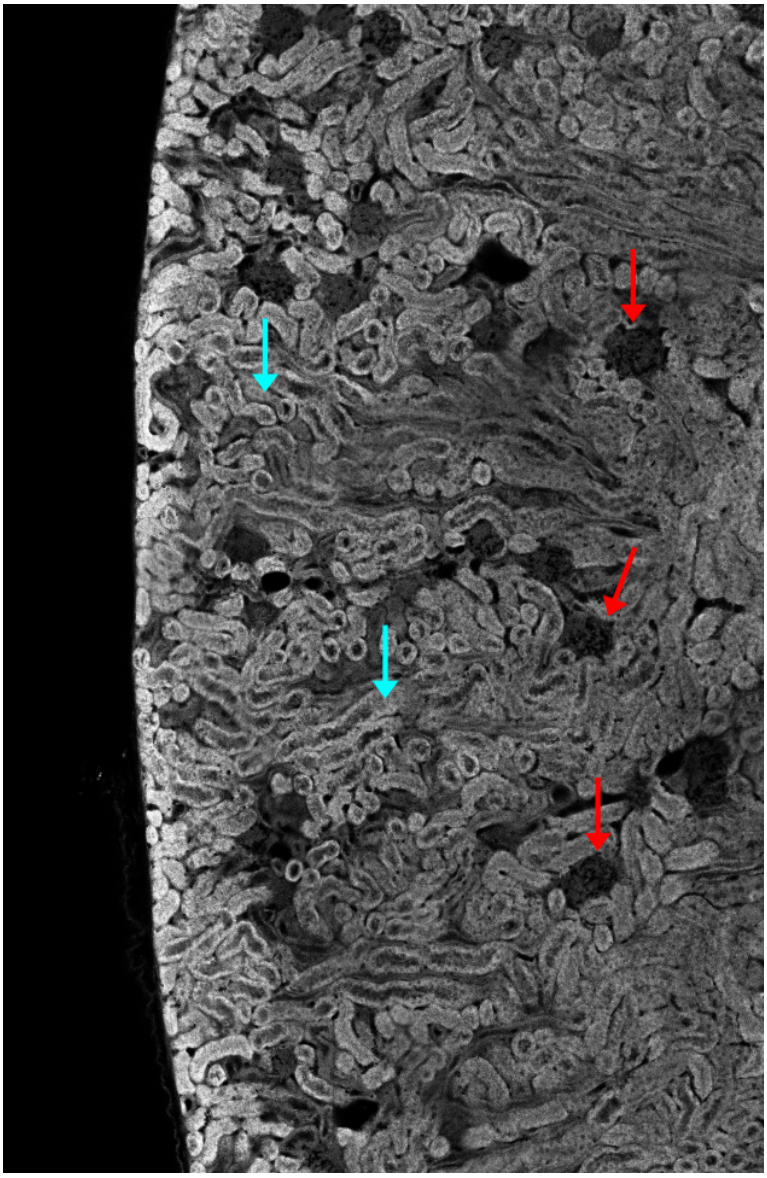

Figure S4. Fluorescent microscopy of kidney's cryosections. Autofluorescence channel (405 nm excitation, 415–470 nm emission detection). Red arrows point to glomeruli, blue arrows point to renal tubules.
